# Core Facility for Supporting Research and Technological Development in Health: A Review of Its Concept and the Brazilian Context

**DOI:** 10.1155/tswj/8865033

**Published:** 2025-12-08

**Authors:** André Browne Ribeiro e Oliveira, Marcelo Santos Ramos, Martha Silvia Martinez-Silveira, Claudio Damasceno Pinto, Cristiano Vasconcellos Ferreira, Bruna Aparecida Souza Machado

**Affiliations:** ^1^ SENAI CIMATEC University, Salvador, Bahia, Brazil; ^2^ Gonçalo Moniz Institute (IGM) Oswaldo Cruz Foundation (Fiocruz), Salvador, Bahia, Brazil; ^3^ Oswaldo Cruz Institute (IOC) Oswaldo Cruz Foundation (Fiocruz), Rio de Janeiro, Rio de Janeiro, Brazil; ^4^ Department of Civil Engineering, Federal University of Santa Catarina, Florianópolis, Santa Catarina, Brazil, ufsc.br

**Keywords:** core facility, multiuser laboratory, public research institutions, research infrastructure

## Abstract

Core facilities are important infrastructures that support scientific research, technological development, and innovation in health. Their concept has evolved in recent decades, incorporating new characteristics and functionalities that highlight their importance in scientific field. In Brazil, these units gained relevance in the late 1990s, accompanying the expansion of science, technology, and innovation policies in health. The objective of this study was to investigate the concepts of core facilities present in the literature, understand their insertion in Brazilian context, propose categories of conceptual analysis for core facilities, and define a concept that encompasses the main characteristics of these multiuser research support units. To this end, an analytical review was conducted through an extensive search for documents published between 1990 and 2024, involving scientific articles, technical–managerial documents, and research funding notices. The various definitions of core facility were evaluated, their main characteristics mapped, the Brazilian context assessed, eight categories of conceptual analysis identified and described, and, based on these, a new concept for these structures proposed. The results contribute to scientific literature and may be valuable for both researchers and science and technology managers in understanding their scope of action, in inferring their impact on biomedical sciences, and in building a management model appropriate for core facilities that support scientific research in the health field.

## 1. Introduction

Modern science is based on a collaborative ecosystem with shared resources, which involves partnership networks between technology experts and researchers capable of aligning all the necessary elements for technological mastery, including the full use of technology, risk reduction, implementation of new scientific techniques and protocols, service provision, evaluation, and complementation of skills located outside the organizational boundary. The core facility environment is the main structure for supporting this approach [1, 2].

From this perspective, since the late 1960s, there have been records of scientific research institutions that began to make large pieces of robust and sophisticated equipment available in centralized laboratories, with the aim of distributing the costs of equipment and highly qualified personnel among as many research groups as possible. It is important to note that this institutional arrangement has proved advantageous by reducing barriers to academic–scientific activities and the intellectual fertilization that has significantly enriched research in the institutions that have adhered this approach [3].

The structural model of science, technology, and innovation institutions, characterized by the allocation of researchers to a laboratory equipped with robust, sophisticated equipment for the almost exclusive use of their team, has been gradually changing, and centralized research support infrastructures, known as core facilities, have been gaining increasing relevance since the 1980s [4]. These centralized, multiuser research support structures now support more competitive science by providing specialized technical and scientific services, highly complex technological equipment, and human resources with a high level of technical expertise [1]. In Brazil, the presence of these laboratories has also been recorded since the 1980s, specifically from the experience of induction and promotion by the National Council for Scientific and Technological Development [5, 6].

Core facilities are becoming increasingly important in universities, research institutes, and commercial laboratories, due to the continuous advancement and sophistication of the equipment needed for scientific work [7]. Scientific research has a high cost of purchasing and maintaining equipment, which is no longer supported by individual research laboratories, especially when they are part of research institutes made up of several laboratories [7]. In this way, these infrastructures facilitate more efficient research and help reduce redundant investment in infrastructure needed to conduct competitive and relevant scientific research [1].

Science and technology are constantly evolving, and the big challenge for core facilities is to remain agile and continuously adapt in a constantly changing research environment [2]. Core facilities therefore depend on an enabling institutional structure, the foundations of which are long‐term strategic planning for investments in research, development and innovation infrastructure, technological tools, and the development of competencies and skills of its human resources, as well as closer collaboration between other institutional players who have an interest in this research support infrastructure [8].

Scientific techniques are constantly evolving, making research more complex and requiring the integration of different methods, which in turn require specific specialized knowledge. In addition, the grant application process for complex research has become more rigorous and competitive, requiring the use of advanced tools and methods to ensure that the issues and challenges of the study are adequately addressed [9].

In this context, core facilities are a strategic advantage in the portfolio of academic and scientific institutes, acting as catalysts for the construction and advancement of an efficient research and innovation environment [10]. This model facilitates user access to a growing range of cutting‐edge technologies while optimizing the management of research projects, reducing costs, and promoting the efficient use of resources [11]. Although it is a relatively new concept, core facilities have received significant attention due to the high return on investment they provide, especially given the high costs of acquiring and maintaining equipment and the level of expertise required for its proper use and management to provide effective support for scientific research [1].

Given the relevance and widespread strategic use of these shared structures to optimize the resources applied to research, technological development, and innovation, this study is aimed at evaluating the various aspects that characterize the concepts of core facilities present in the scientific literature dedicated to the topic, evaluate these research support infrastructures in Brazilian context, propose categories of conceptual analysis that characterize core facility, and define a concept that encompasses the main characteristics of this type of research infrastructure in health area, based on the various definitions presented in the selected and analyzed documents.

This study contributes to scientific literature by providing an update and better understanding of the concept of core facilities in health research and may be important for both researchers and RD&I managers by harmonizing the various approaches presented in the literature on the subject. Thus, based on the definition of specific categories of analysis for these research support infrastructures, it will be possible to identify the main aspects to be evaluated in the operations of these units, in order to strengthen understanding of their characteristics, disseminate implementation and management practices, and enable the expansion and demonstration of the impact of these support infrastructures on institutional results.

## 2. Material and Methods

The methodology used in this article involved an analytical review of the concept of core facility in health research, with a wide‐ranging search for articles, visits to websites and open‐access documents from science, technology, and innovation institutions, universities, and research funding agencies, with no limitations as to the type of document analyzed in order to guarantee a broad mapping. A search was carried out in scientific literature sources where articles in international and national literature were identified, as well as public or publicized institutional documents such as ordinances, regulations, regiments, notices, and resolutions.

The following scientific literature sources were used: PubMed and Google Scholar. PubMed was chosen because it is a platform that brings together the Medline database and another wide selection of scientific articles in health area, with more than 38 million records, making it the most complete database of information in medicine and health. The research was expanded using Google Scholar, as it is a search engine specializing in scientific and academic literature covering a wide range of topics and offering the possibility of locating national studies not indexed in PubMed. Once the articles had been selected, the works cited were tracked down in order to further expand the search. In addition, websites cited in the works were also accessed, where other studies of interest could be located.

Considering the difficulty of identifying the concept of a core facility in healthcare in Brazil in database searches, the broad search procedure was justified in order to ensure that a greater number of existing documents could be analyzed. Thus, in addition to databases, websites of scientific, technological, and innovation institutions (ICTs), universities, and development agencies, as well as the National Research Infrastructure Platform—MCTI, were consulted to identify multiuser laboratories in the health field in Brazil.

The PubMed search was conducted in April 2024, with no restrictions on the publication period of the articles, in order to obtain as many documents as possible. The search strategy adopted was defined with the support of a specialized librarian to ensure sensitivity, specificity, and comprehensiveness of the results: “core facilit ^∗^”[Title] OR centralized facility ^∗^“[Title] OR (“multi user ^∗^“[Title] AND (equipment∗[TIAB] OR facilit∗[TIAB])) OR “shared facilit ^∗^”[TI] OR “sharing facilit ^∗^”[TI] OR “sharing equipment” [TI]. This wording was chosen because it encompasses the various terms used internationally to describe these research support infrastructures.

On Google Scholar, the search was also conducted in April 2024, covering the period from 1999 to 2024, using the terms in Portuguese: “technological platform” and “Fiocruz” anywhere in the text, resulting in 497 documents on various topics. This combination was chosen to identify national documents directly related to institutional experiences in the Brazilian context. The correct form of the terms was tested beforehand to ensure the retrieval of results. Another aspect to be considered is that there is no specific term in the Brazilian context to identify these research support infrastructures in the health area, so the use of terms such as “multiuser units” and/or “technology platform” expands the search too much, making the research unfeasible.

The documents were selected based on the following inclusion criteria: articles and documents that directly addressed the concept, the definition, or characteristics of core facilities in health field; publications in Portuguese or English; open access or institutionally available texts; and normative, regulatory, or financing documents related to the topic.

The following exclusion criteria were adopted: articles written in languages other than English or Portuguese, duplicate articles, articles that were unrelated to the topic or irrelevant to the research, and documents that did not present a definition or characterization of a core facility.

The screening of documents was carried out independently by two researchers. First, titles, abstracts, and keywords were read in order to establish the relevance and categorization of publications focusing on the object of investigation of this study through the application of inclusion and exclusion criteria. Disagreements between reviewers were resolved by consensus, without the need for intervention by a third evaluator. A spreadsheet was thus created, recording year of publication, type of document, and the existence of a concept, definition, or characteristics of core facilities in health field, with the respective transcription.

The complementary search steps were divided into tracking references, consulting websites cited in the articles, searching for national technical documents, and surveying funding announcements. Initially, the reference lists of the selected articles were analyzed to identify new works. Direct access was also made to institutional pages indicated in the articles, resulting in the inclusion of three additional documents: a proposal for action for an alliance of core facilities, a study on the impact of these platforms on research, and an executive summary.

The national technical management documents were identified by conducting a search for multiuser laboratories on the National Research Infrastructure Platform—MCTI, which aims to map and gather information on the research infrastructure of scientific, technological, and innovation institutions (ICTs) in Brazil. This platform located three universities and one research foundation with multiuser structures in health area compatible with this study: the Federal University of Espírito Santo, the Federal University of Uberlândia, the Federal University of Santa Catarina, and the Oswaldo Cruz Foundation. These documents are openly accessible on the respective websites of these universities and ICT.

Based on the objectives of this study, a search was conducted on the websites of the main funding agencies to identify specific calls for proposals for the implementation of multiuser laboratories. Thus, three public calls for proposals were identified to support multiuser scientific and technological research infrastructure: one at federal level by the Brazilian Innovation Agency (FINEP), called “Public Call MCTI/FINEP/FNDCT/CT‐INFRA/National Multi‐user Centers 2022,” and two at state level, by the São Paulo Research Foundation (FAPESP), launched in 2022, “Call for Multi‐user Equipment for Scientific Use – 2022” and by the Minas Gerais State Research Support Foundation (FAPMIG), “Call FAPEMIG 04/2023 Technology and Infrastructure Centers for Research at UEMG and UNIMONTES.”

In order to define the conceptual analysis categories that characterize core facilities, an analysis was carried out of different concepts presented by different authors, by identifying the main approaches and aspects related to the topic in each text. In this way, the main characteristics used to define a core facility were classified. Each characteristic identified was carefully grouped based on its similarities and meanings, with the aim of allowing common elements to be brought together in a simplified representation.

**Figure 1:**
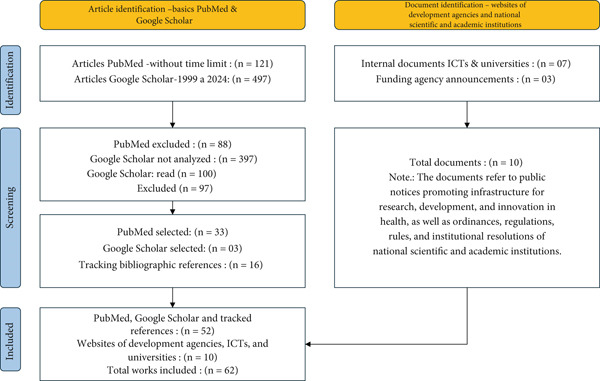
The process of identifying, selecting, determining the eligibility of, and including studies (based on the PRISMA method). *Source:* Prepared by the authors.

The process of identifying, selecting, determining the eligibility of, and including studies was systematized using a diagram based on the PRISMA method (Figure 1), which quantitatively presents the steps and number of documents in each phase of the literature review.

## 3. Results

### 3.1. Descriptive Statistics on the Literature Review

Originally, 121 publications were found on PubMed, and after reading the titles, abstracts, and keywords, 88 articles were excluded, and 33 were picked for review. A comprehensive analysis of these works allowed for the incorporation of 13 additional bibliographic references, located by tracking citations from the previously selected articles, totaling 46 articles from PubMed. In the expanded search on Google Scholar, the first 100 occurrences were analyzed. The adoption of this procedure is justified by the greater probability of relevance of the results displayed on the first pages of this database, according to its own ranking algorithm, and by the lack of a controlled descriptor in Portuguese capable of adequately delimiting the term core facility. In this search, three articles related to the topic were identified, and three more articles were added, located in the tracking of works cited by the authors of the first selected articles, thus totaling six articles from this database. Thus, the set of scientific publications in this study consists of 52 articles.

Table 1 shows the diversity of the documents analyzed, classifying them by type and origin, and demonstrates the importance of the topic, especially in international literature, which accounts for 79% of the documents reviewed, while 21% of the documents analyzed were published in Brazil.

**Table 1:** Quantitative and percentage distributions of documents included and analyzed in this narrative literature review.

**N**	**Document type**	**Total**	**%**
1	International journal articles/foreign authors	46	74%
2	National periodical articles/Brazilian authors	6	10%
3	National internal document	4	6%
4	International internal document	3	5%
5	National funding notice	3	5%
	Total	62	100%

*Note:* Source: Prepared by the authors, based on information obtained in the analytical review, in April 2024.

**Figure 2:**
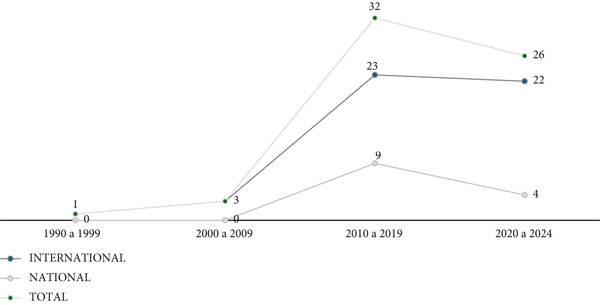
Quantitative distribution of documents included in the review, segmented by the decade in which they were published between 1990 and 2024 (*N* = 62). *Source:* Prepared by the authors, based on information obtained in the analytical review, in April 2024.

With regard to the time distribution of the findings of this analytical review, documents published between 1999 and 2024 were identified. The documents were grouped by decade in order to demonstrate the conceptual evolution of core facilities. This division by four decades shows the construction of the concept of core facilities in health over time and explains the increase in interest in the subject, since only one document was identified at the end of the 1990s and in the decade from 2010 to 2019, this number reached 32 works, which represents 51.6% of the total documents studied, and only in the first 4 years of the 2020s, there are already 26 documents, which make up 41.9% of the total, as shown in Figure 2.

Figure 2 also shows that the subject has been covered in international literature since the 1990s and, up to May 2024, 49 documents and papers on core facilities were included, which represents 79% of the texts analyzed. In relation to the national literature review (Brazil), the first documents appeared more than 10 years later, from the 2010s onwards, and totalized only 13 bibliographical references in the period, which constituted 21% of the 62 items that made up the material analyzed.

### 3.2. The Conceptual Evolution of Core Facilities to Support Research, Technological Development, and Innovation in Health

Core facilities are an integral part of the life sciences research universe, as providers of centralized access to technological resources and expertise, with one of their main strategic objectives being to serve as a knowledge incubator [12, 13]. These research infrastructures are recognized as the main drivers of research in research institutes and must be able to remain agile and constantly adapt to the latest advances in science [2].

The huge increase in projects requiring advanced technologies has required the creation of state‐of‐the‐art core facilities. Initially, their main function was to favor access to sophisticated equipment with a high acquisition cost, either as an open‐access facility or by generating data as a service [14]. Over the last 10 years, the focus has shifted to a full‐service model, where the hub generally acts as a consultant for an entire experimental workflow. Today, core facilities are also asked to innovate to create methods and applications together with researchers [14].

**Figure 3:**
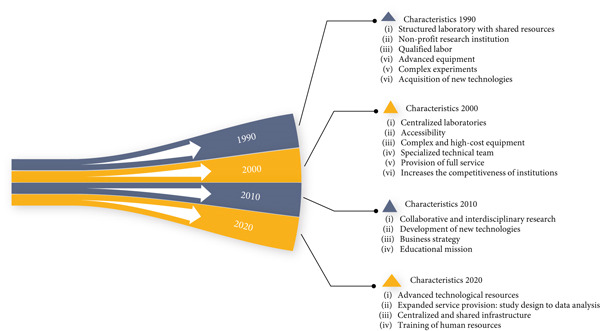
Conceptual evolution of core facilities: main characteristics identified between 1990 and 2020. *Source:* Prepared by the authors, based on information obtained in the analytical review, in April 2024.

Figure 3 shows the conceptual evolution of core facilities over the last four decades, highlighting the main characteristics found in each decade.

In the 1990s, the core facility was defined as laboratories with shared resources, in nonprofit research institutions, with two essential components: a highly qualified workforce and advanced equipment, which allows their institutions to have a competitive advantage in the search for resources for their research [15]. The technical staff of these laboratories make complex experiments possible, creating the possibility of acquiring new methodologies. This definition of a core facility platform is cited by other authors in five articles written in the 2010s.

Based on articles published in the 2000s, core facilities are now defined as centralized or decentralized laboratories, accessible to all the organization, which have complex and expensive equipment, with technical staff specialized in data acquisition and interpretation, providing comprehensive services, maintaining equipment, and training users. Research institutions now recognize that core facilities are a means of providing advanced technologies that maintain or improve institutional competitiveness [3, 16, 17].

Since 2010, core facilities have been characterized not only with the concept of the previous decade but also with the inclusion of aspects related to collaborative and interdisciplinary research [18–20], the possibility of developing new technologies, with the potential to develop new uses for existing technologies [18], and the need to act with a business strategy, with professional management, in order to guarantee greater financial sustainability [20–22], and it starts to highlight the mission of education, related to training the next generation of scientists [23–25].

In the 2020s, the concept of core facility becomes more comprehensive, with the participation of all the conceptual analysis categories proposed in this study. Thus, these research infrastructure units are characterized by a set of sophisticated and shared technological resources (equipment, technology, and human resources) in a centralized way, with a broad service provision, helping researchers from research formatting to data analysis, as well as training new users and the process of training human resources for scientific research.

It strengthens the collaborative and interdisciplinary research stance and is an ideal environment for developing and testing new technologies quickly and with greater accessibility to the research community [2]. Therefore, in order to operationalize these structures, they are increasingly acting with a dual nature of laboratory and small business, seeking to optimize these resources [22, 26].

### 3.3. Identification and Description of Conceptual Analysis Categories for Core Facilities to Support Scientific Research in Health

The literature review allowed us to identify the main characteristics of core facilities, which formed the basis for defining the conceptual analysis categories proposed in this study. These represent dimensions used to study and understand the functioning and structure of core facilities in a more comprehensive manner.

Each concept was developed based on the identification of recurring terms, expressions, and quotes in the analyzed documents, directly related to the conceptual analysis categories. To operationalize this process, the two researchers read each document in its entirety and recorded the keywords, characteristics, and relevant aspects identified in a spreadsheet (Microsoft Excel). The extracted characteristics were then grouped by similarity and common terms, organizing them in descending order—from the most comprehensive category to the least comprehensive or those that did not fit into any other category.

This procedure resulted in the identification of 49 distinct characteristics. For inclusion in a conceptual category, its minimum occurrence in two or more documents was considered, associated with conceptual relevance for understanding the concept of core facilities, even if infrequent. As an example, the category “development of new technologies and techniques” was retained, despite appearing in only 22% of publications, as it represents a strategic aspect for scientific advancement.

After joint analysis and discussion among the researchers, the 49 characteristics were grouped into eight categories of conceptual analysis, which served as axes for aggregating similar elements. These categories, together with their descriptions and percentages of incidence in the reviewed literature, are presented in Table 2.

**Table 2:** Categories of conceptual analysis, their descriptions, and percentage distributions of citations in the reviewed literature.

**N**	**Categories of conceptual analysis**	**Conceptual analysis category descriptions**	**No. of articles**	**%**
1	Equipment and technologies (A)	Refers to the equipment and technologies made available that are characterized by being sophisticated, specialized, expensive, advanced, exclusive, innovative, complex, high performance, state of the art and that can be used for more than a variety of applications, and must be kept up to date	48	77.42%

2	Service provision (B)	It refers to the way in which core facilities operate by providing research support services in a complete, comprehensive, and specialized manner: from experimental design (designing experiments) to the analysis and processing of results, such as specialized consultancy (the best way to use available resources to address different research questions), collaboration, user training, and the mission of education and training of human resources, available to contribute to all requests	46	74.19%

3	Centralized and shared resources (C)	It refers to the availability of a state‐of‐the‐art research infrastructure, centralized in a single physical unit, for the entire institution, enabling unrestricted access, including for external public or private institutions	44	70.97%

4	Specialized technical team (D)	Refers to the human resources responsible for operating the units called core facilities that must have highly qualified, competent, trained, experienced labor, specialized professionals (specialists), with specific knowledge, with the ability to provide services, knowledge for equipment maintenance, user training, human resource training, and the ability to develop and acquire new methodologies	38	61.29%

5	Economy (E)	It refers to the creation of research support infrastructures available to the entire institution, enabling greater competitiveness in research, in the submission of notices, in the search for resources, and in carrying out research quickly and economically, with greater efficiency. These units enable the optimization of resources, avoiding equipment being purchased twice and optimizing use with greater bargaining power for the purchase of supplies and equipment maintenance	22	35.48%

6	Collaboration and interdisciplinarity (F)	It refers to the need for life science research to combine different approaches, as well as the impossibility of a single research group mastering all techniques, and these structures must provide and maintain all the technologies necessary for interdisciplinary approaches. These research infrastructures become a facilitator/catalyst for new interactions between institutions, always focused on collaboration	17	27.42%

7	Business attitude (G)	It is characterized by the way these units operate, having a dual function as a research laboratory and a nonprofit company. The services provided are charged in order to cover their operational costs, acting as a contact zone with the private sector. From this perspective, in addition to technical and scientific skills, these units must understand customer service, quality assurance, and business administration (managing budgets, marketing, and business plans)	17	27.42%

8	Development off new technologies (H)	It refers to the ability to effectively explore the scientific value and market potential of new technologies, the ability to develop new technologies and research techniques, and the ability to be an incubator for new or immature technologies	14	22.58%

*Note:* Source: Prepared by the authors, based on information obtained in the analytical review, in April 2024.

Detailed information on the 62 documents included in the review, titles, authors, document type, and year of publication, are available in Table S1. Among the documents analyzed, 52 were scientific papers, 7 were internal documents, and 3 were research funding announcements. The last eight columns on the right show the categories of conceptual analysis, which were classified as follows: (A) equipment and technologies, (B) service provision, (C) centralized and shared resources, (D) specialized technical team, (E) cost‐effectiveness, (F) collaboration and interdisciplinarity, (G) entrepreneurial attitude, and (H) development of new technologies and techniques [27–47]. Scientific papers [48–68] deal with the theme of core facility, where at least one of the categories of analysis was identified. These papers comprise the study and are listed in Table S1 of the supplementary material.

Table S1 also shows that none of the 62 documents covered all eight categories simultaneously. It was found that 25.8% of the documents defined core facilities based on four distinct categories, 37.1% used up to three categories, and another 37.1% used between five and seven categories to characterize centralized multiuser units supporting scientific research in health.

#### 3.3.1. Equipment and Technology

The equipment and technology analysis category is present in all decades analyzed and was identified in 48 documents, which corresponds to 77.42% of the literature reviewed, demonstrating that the reason for the existence of a core facility is a provide cutting‐edge technology to promote and facilitate the development of a competitive research environment within institutions, that is, to have a concentration of technology [5, 8, 20, 48].

In the Brazilian context, the findings follow the same trend. Among the 13 national studies examined, only one does not mention this category, representing 92.31% of publications. It is also observed that, starting in 2020, there was an intensification in the offer of funding calls aimed at expanding the multiuser equipment park in the country, through the structuring and strengthening of core facilities [49–51].

#### 3.3.2. Service Provision

The service provision analysis category, which ranks second in citations with presence in 74.19% of the 62 publications analyzed, is related to the way in which core facilities operate through the provision of research support services. Service provision has been highlighted as an analysis category from the 2000s, covering the delivery of results and user training for basic equipment operation [3].

However, the issue related to education, with a focus on training human resources for the research area, was presented from the 2010s onwards, as well as the expansion and qualification of the provision of the service also involving consultancy on the experimental design of the research [19, 23–25, 52] and is addressed again in the 2020s [12, 53, 54].

In the Brazilian literature analyzed, the category of service provision has been present since the 2010s, constituting a central characteristic. Core facilities are described as units that meet both internal and external demands, playing a strategic role in strengthening scientific research in the country [5, 48–51, 55, 56].

#### 3.3.3. Centralized and Shared Resources

The analysis category of centralized and shared resources was cited in 44 (70.97%) of the 62 documents analyzed, making it the third most frequent in the literature. This conceptual category has been present since the 1990s, related to resource sharing [15], which began to have a connotation of accessibility in the following decades.

Centralization of resources expands knowledge on core facilities, avoiding duplication of services, reducing the administrative burden, and increasing the impact of these units on institutional objectives, which fits in with the vision of centralizing infrastructure as a means of disseminating science‐based knowledge [57, 58].

In Brazil, the aspect of infrastructure sharing was identified since the first concepts analyzed in the 2010s, but the aspect related to the centralization of these units was initially treated as technological concentration [5] and then as centralized infrastructure. From 2020 onward, nation calls for proposals for funding require an infrastructure that can be shared with internal and external users, serving several research groups, to be eligible for funding [5, 48–51, 55, 56].

#### 3.3.4. Specialized Technical Team

This category of analysis is present in all decades and was described in 61.29% of the bibliographic and documentary material analyzed. A specialized technical team is necessary to have qualified human resources, from equipment operation to labor training. Without the proper qualifications and experience, it is not possible to guarantee that the technology and/or equipment will be used to its full potential [8].

In the 1990s, the technical team was mainly characterized as providing support for complex experiments, focusing on developing skills for operating equipment [15]. From the 2000s onwards, the expansion of service provision demanded new skills, including the ability to select the most effective methods for research objectives, interpret data, and work in multidisciplinary contexts [3, 16]. In the 2010s, the educational dimension gained prominence, demanding the need to develop skills linked to the training of qualified human resources in research, technological development, and innovation in health [8, 18, 59].

In the Brazilian context, the initial focus in the 2010s was based on the technical competence necessary to conduct trials and expand analytical capacity [48]. From the middle of the decade onwards, the importance of the educational role of teams was highlighted, aimed at training users and expanding their autonomy in research development [52, 60]. Starting in 2020, the emphasis has been on expanding skills related to the handling, maintenance, and conservation of equipment, as well as support in planning, conducting experiments, and analyzing data [53].

#### 3.3.5. Economy

This category of analysis is present in 35.48% of the studies. This category refers to the reduction of the cost of access to technology and equipment, in the search for greater efficiency [17, 23, 24, 61]. Core facilities seek to optimize resources, avoiding duplicate equipment purchases, in addition to enabling greater negotiating power for ICTs for the purchase of inputs and equipment maintenance [62].

The issue related to cost‐effectiveness, although it has been addressed since the 1990s, began to be more emphasized from the 2010s onwards, due to the slowdown in the main economies, in the period between 2008 and 2019 [63].

In the Brazilian context, this category appears in 30.77% of the documents analyzed, standing out in the search for optimization of investments in highly complex multiuser infrastructure. The focus is on rationalizing financial, material, and human resources in order to maximize their use and reduce usage and maintenance costs [51–53, 55].

#### 3.3.6. Collaboration and Interdisciplinarity

This category of analysis was found in 27.42% of the references and refers to the need to combine different approaches in life science research, which has led to core facilities acting as a facilitator/catalyst for new interactions between research institutions. Through collaboration, experiences and expertise are shared, including between institutes, as a way of remaining competitive at the forefront of life science, and this is a hallmark of biomedical research [8, 9, 18, 19, 64].

The issues of collaborative research and interdisciplinarity have become themes in the descriptions of core facilities since the 2010s, with the creation of new disciplines in the area of biology and the short lifespan of new technologies [18].

In Brazil, multiuser units stand out for their multidisciplinary vocation, functioning as environments conducive to the implementation and management of interdisciplinary projects in basic and applied research [53, 54, 65].

#### 3.3.7. Business Attitude

The conceptual analysis category business posture is present in 17 (27.42%) of the 62 documents studied. It involves the way core facilities operate due to the current restrictive economic scenario that requires the optimization of the existing budget, imposing on them a dual function as a research laboratory and a nonprofit company. Thus, the services provided are charged for in order to cover their operational costs [10, 15, 20, 22, 26].

From this perspective, in addition to technical and scientific skills, these units must seek greater operational efficiency and competitiveness in research, which is a differential in the search for financial resources [15, 58]. In this sense, it is necessary to adopt a business strategy that develops skills to improve user service and ensure quality in service delivery. To this end, it is essential to incorporate concepts of business administration (budget management, marketing, and business plan) and strategic planning [21, 26, 66].

The issue of having a business vision was initially focused on the idea of being competitive in attracting financial resources [15]. However, in the 2010s, units began to be concerned with professionalizing their operations, incorporating business practices, in an attempt to better organize themselves and demonstrate the importance of their performance for the institution [21, 58, 66].

In Brazil, corporate stance appears to be mainly linked to institutional strategic plans, which define specific objectives and targets to be achieved [67].

#### 3.3.8. Development of New Technologies and Techniques

The conceptual analysis category development of new technologies and techniques was identified in 14 publications studied, which represented the lowest percentage of citations with 22.58% of the 62 documents analyzed. It was initially treated as the ability to attract new technologies, but this characteristic began to stand out again in the 2010s, demonstrating the importance of developing immature technologies in an initial state and the ability to present new ways of using technology through the development of new techniques [14, 18, 22, 55].

In Brazil, this category manifests itself through the provision of services to technology‐based companies, using platform infrastructure to stimulate research, technological development, and innovation processes [49].

#### 3.3.9. Summary of Analysis Categories

**Figure 4:**
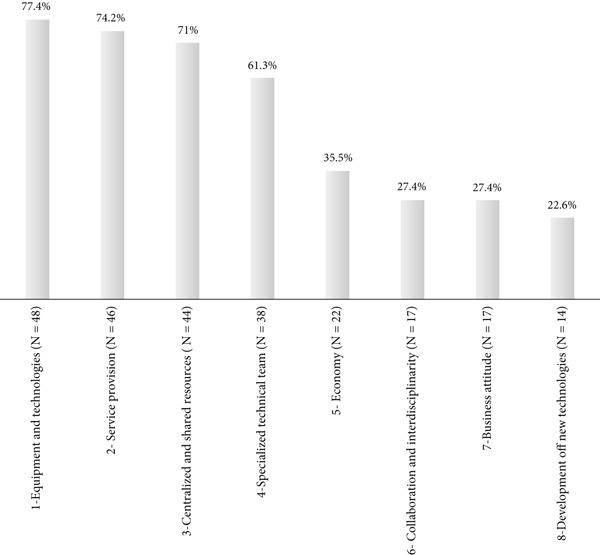
Percentage distribution of incidence of conceptual analysis categories identified in the literature review on the concept of core facility (*N* = 62). *Source:* Prepared by the authors, based on information obtained in the analytical review, in April 2024.

In order to briefly consolidate the results found in this section of the work, Figure 4 demonstrates the degree of relevance of each of the eight categories of conceptual analysis identified in the literature review, as it visually presents the percentage of incidence of each of them in relation to the total number of articles to be analyzed (*N* = 62) and highlights the aspects that underpin the definition of what a core facility is. This concept is strongly related to the structuring of support units for research, technological development, and innovation in health. It is characterized by the presence of sophisticated, complex, high‐cost, and high‐performance equipment and technologies, made available by ICTs in a centralized and shared manner. This approach enables access for the entire institution and allows for the extramural use of these structures, thereby maximizing the results of local and regional science, technology, and innovation systems in health. This organizational model expands the scope of the provision of support services for scientific research in health, through a specialized technical team and the optimization of resources made possible by the adoption of a business management approach and the encouragement of collaboration and interdisciplinarity, which form a fertile substrate for the development of new technologies in the field of biomedical sciences.

### 3.4. The Concept of a Core Facility in Brazil

In Brazil, although there have been records of the existence of core facilities since the 1980s, documents formally presenting a concept have only been identified since 2011. As an example, the Oswaldo Cruz Foundation (Fiocruz) had its first core facilities implemented in 2004 and its standards and guidelines, with the formalization of a concept, in 2015 [5, 60].

In the documents analyzed from the 2010s, which included six articles and three internal documents (internal regulations, internal rules, and internal ordinances), characteristics were identified that make it possible to conceptualize core facilities as follows: a set of multiuser laboratories operating in a centralized way, offering shared and dedicated use with greater accessibility. These facilities provide services with advanced, high‐cost equipment and technology and employ qualified technical staff to operate the equipment, support research activities, train human resources, and facilitate interaction with other higher education institutions, research institutions, and the business sector in public and private spheres, in line with the institutional strategic plan. These structures make it possible to optimize public resources, improve the quality and competitiveness of research projects, develop interdisciplinary projects, and aim to contribute to regional technological development.

The characteristics that make it possible to conceptualize the core facilities identified in the national documents analyzed are shown in Table 3.

**Table 3:** Main features found in national documents.

**N**	**Features**	**Authors**
1	Multiuser/centralized/shared use laboratory	[5, 48–53, 55, 56, 60, 65, 67]
2	Accessibility	[52, 55, 56, 60, 65]
3	Advanced and high‐cost equipment	[48, 49, 53, 55, 56, 60, 65]
4	Specialized technical team	[5, 48–53, 55, 60, 68]
5	Educational mission	[50–53, 55, 60]
6	Interaction with other public and private institutions	[5, 49–51, 53, 55, 65, 68]
7	Aligned with the institutional strategic plan	[56, 67]
8	Service provision	[5, 49, 52, 53, 55, 56, 60, 67, 68]
9	Regional technological development	[49–51, 53]
10	Collaborative and interdisciplinary research	[51, 53]

*Note:* Source: Prepared by the authors, based on information obtained in the analytical review, in April 2024.

In the 2020s, the documents analyzed were notices from funding agencies and the internal regulations of a federal university′s core facility, which added the following characteristics in relation to the previous decade: the impact of the work of these research infrastructures on regional technological development and encouraging the implementation of collaborative and interdisciplinary projects [49–51, 53].

## 4. Conclusion

This literature review contributed to broadening the understanding of the characteristics of core facilities supporting scientific research in health, which gained greater relevance from the late 1990s onwards.

One limitation is that the study did not adopt a systematic review as a methodological strategy. This decision was due to the difficulty of parameterizing the terminology in Portuguese, given the diversity of similar terms that would result in overly broad searches with low precision. This limitation may have led to the exclusion of some relevant publications. To mitigate this risk, complementary search steps were incorporated, including tracking references, consulting websites cited in the articles, analyzing technical documents, and surveying funding announcements.

The studies analyzed first emphasize the optimization of infrastructure and human resources to reduce investment costs in product and process technologies. In the first decade of the 2000s, there was greater concern about the competitiveness of universities and ICTs in a collaborative and interdisciplinary environment, which demanded the provision of more complex and sophisticated services. From 2020 onwards, a new dimension came to the fore, related to the impacts generated by the implementation, sustainability, and strengthening of centralized and shared use infrastructures to support scientific research. Since then, this organizational model has begun to measure and evaluate results that go beyond its operational and routine activities, as it also aims to contribute to the training of qualified human resources, the development of new technologies, and the establishment of collaborative and interdisciplinary networks that impact scientific and technological advances in health.

In addition, this study contributes to mapping and describing eight categories of conceptual analysis that characterize core facilities. These units can be defined as infrastructures that support scientific research, technological development, and innovation in health, offering shared access to advanced equipment, cutting‐edge technologies, and qualified labor, centralized in research institutes. They provide specialized services, including experimental design, data analysis, consulting, collaboration, training, and human resource development, based on management tools that ensure efficiency and sustainability.

In Brazil, core facilities follow global trends, with an emphasis on the expansion of multiuser infrastructures, the promotion of resource sharing, technical and educational training, and strategic coordination to optimize investments. Recent calls for proposals reinforce these standards, requiring core facilities to be accessible, multiuser, and capable of serving both internal and external researchers.

In summary, the findings show that core facilities not only provide infrastructure and services but also act as strategic environments that promote training, innovation, collaboration, and efficiency, consolidating themselves as central instruments in the advancement of scientific research in health.

Finally, based on the conceptual analysis categories proposed in this study, it will be possible to identify the main aspects that should be evaluated in relation to the operation of these units, contributing to the proposal of performance evaluation indicators aligned with each of the eight categories, as well as guiding the construction of a management model appropriate for core facilities supporting scientific research in the biomedical area.

## Ethics Statement

The authors have nothing to report.

## Consent

The authors have nothing to report.

## Conflicts of Interest

The authors declare no conflicts of interest.

## Author Contributions

Conception and design of the study: André Browne Ribeiro e Oliveira, Claudio Damasceno Pinto, Marcelo Santos Ramos, Cristiano Vasconcellos Ferreira, and Bruna Aparecida Souza Machado; data acquisition, analysis, or interpretation: André Browne Ribeiro e Oliveira, Claudio Damasceno Pinto, Marcelo Santos Ramos, Cristiano Vasconcellos Ferreira, and Bruna Aparecida Souza Machado; manuscript editor: André Browne Ribeiro e Oliveira, Claudio Damasceno Pinto, Marcelo Santos Ramos, Cristiano Vasconcellos Ferreira, and Bruna Aparecida Souza Machado; critical review of intellectual content: Claudio Damasceno Pinto, Cristiano Vasconcellos Ferreira, Bruna Aparecida Souza Machado, and Martha Silvia Martinez‐Silveira.

## Funding

No funding was received for this manuscript.

## Supporting information


**Supporting Information** Additional supporting information can be found online in the Supporting Information section. Table S1: List of documents included in the literature review on core facilities in health research, indicating titles, authors, document types, publication years, and conceptual categories (A–H).

## Data Availability

Data sharing is not applicable to this article as no datasets were generated or analyzed during the current study.
