# Supplementary material for: Core Facility for Supporting Research and Technological Development in Health: A Review of Its Concept and the Brazilian Context
Source: ScientificWorldJournal. 2025 Dec 8;2025:8865033. doi: 10.1155/tswj/8865033 (PMC12767582; doi:10.1155/tswj/8865033)
Supplement: Supplementary file 1 — Supporting Information Additional supporting information can be found online in the Supporting Information section. Table S1: List of documents included in the literature review on core facilities in health research, indicating titles, authors, document types, publication years, and conceptual categories (A–H). [file TSWJ-2025-8865033-s001.zip › SM table S1.docx]

# **Supplementary materials**

# **Table S1**

**Table S1.** Mapping conceptual analysis categories on core facility in the revised documentation.

| **N** | **Title** | **Document type** | **Author** | **Year** | **Conceptual Analysis Categories**  **identified in each of the reviewed documents** (1=Yes, /0=No) | | | | | | | | |
| --- | --- | --- | --- | --- | --- | --- | --- | --- | --- | --- | --- | --- | --- |
|  |  |  |  |  | **A** | **B** | **C** | **D** | **E** | **F** | **G** | **H** | |
| 1 | Research Technologies: Fulfilling the Promise | Article | [15] | 1999 | 1 | 0 | 1 | 1 | 1 | 0 | 1 | 1 | |
| 2 | Organization, management and operation of contemporary academic mass spectrometry service facilities | Article | [3] | 2000 | 1 | 1 | 1 | 1 | 0 | 0 | 0 | 0 | |
| 3 | A bright but demanding future for core facilities | Article | [16]. | 2005 | 1 | 0 | 1 | 1 | 0 | 0 | 1 | 0 | |
| 4 | A framework for managing core facilities within the research enterprise | Article | [17]. | 2009 | 1 | 1 | 1 | 1 | 1 | 0 | 0 | 0 | |
| 5 | Cost (non)-recovery by platform technology facilities in the Bio21 Cluster | Article | [48] | 2010 | 1 | 0 | 0 | 1 | 0 | 0 | 0 | 0 | |
| 6 | University Multi-User Facility Survey—2010 | Article | [49] | 2011 | 0 | 0 | 0 | 1 | 0 | 0 | 0 | 0 | |
| 7 | Core facilities: maximizing the return on investment. | Article | [38] | 2011 | 1 | 1 | 1 | 1 | 0 | 0 | 0 | 0 | |
| 8 | Institutional management of core facilities during challenging financial times | Article | [41] | 2011 | 0 | 1 | 0 | 0 | 1 | 0 | 1 | 0 | |
| 9 | Challenges for proteomics core facilities | Article | [21] | 2011 | 0 | 0 | 1 | 0 | 0 | 0 | 1 | 1 | |
| 10 | Position Paper Excellence Alliance Core Facilities | Internal Documents | [18] | 2011 | 0 | 1 | 1 | 1 | 0 | 1 | 0 | 1 | |
| 11 | ANNEXES I and III of Resolution No. 24/2011 - CUN - Regulations of the multi-user laboratories of the UFES Health Sciences Center (CCS/UFES) | Internal Documents | [34] | 2011 | 1 | 1 | 1 | 1 | 1 | 1 | 0 | 1 | |
| 12 | The use of technological platforms and their implications for the way research is organized in the field of biomedicine: a preliminary analysis of FIOCRUZ's experience | Article | [5] | 2011 | 0 | 1 | 1 | 0 | 0 | 1 | 0 | 0 | |
| 13 | Multi-user equipment, core-facilities and technological platforms: the evolution of organizational strategies for translational health research. | Article | [46] | 2012 | 1 | 1 | 0 | 1 | 0 | 0 | 1 | 0 | |
| 14 | Technological platforms and research in biomedicine - characterization of local use of global devices. | Article | [47] | 2012 | 1 | 1 | 1 | 1 | 0 | 1 | 1 | 1 | |
| 15 | Technological platforms and research practices in biomedicine - preliminary observations on the use of global devices and local institutions | Article | [27] | 2012 | 1 | 1 | 0 | 0 | 0 | 0 | 0 | 0 | |
| 16 | Best practices for core facilities: handling external customers | Article | [50] | 2013 | 1 | 1 | 1 | 1 | 0 | 0 | 0 | 0 | |
| 17 | Partnership between CTSI and business schools can promote best practices for core facilities and resources | Article | [40] | 2013 | 1 | 1 | 1 | 0 | 1 | 0 | 1 | 0 | |
| 18 | Optimizing institutional approaches to enable research | Article | [4] | 2014 | 1 | 0 | 0 | 1 | 0 | 0 | 0 | 0 | |
| 19 | Standard 01/2015 of the Vice-Presidency of Research and Reference Laboratories - VPPLR | Internal Documents | [39] | 2015 | 1 | 1 | 0 | 1 | 0 | 0 | 0 | 0 | |
| 20 | US National Institutes of Health core consolidation–Investing in greater efficiency. | Article | [51] | 2015 | 1 | 1 | 0 | 1 | 0 | 0 | 0 | 0 | |
| 21 | Core facilities: shared support | Article | [45] | 2015 | 1 | 1 | 1 | 0 | 0 | 0 | 0 | 0 | |
| 22 | Importance of inclusive service for corporate use of university research infrastructure in Japan | Article | [52] | 2015 | 1 | 1 | 1 | 0 | 0 | 0 | 0 | 0 | |
| 23 | Swiss light microscopy facilities: A success story for the last fifteen years | Article | [23] | 2016 | 1 | 1 | 0 | 1 | 1 | 0 | 0 | 0 | |
| 24 | A look at equipment sharing in research institutions in the cities of Rio de Janeiro and São Paulo: the main differences, similarities and challenges observed | Article | [35] | 2016 | 1 | 0 | 1 | 0 | 0 | 0 | 1 | 0 | |
| 25 | Sharing Core Facilities and Research Resources--An Investment in Accelerating Scientific Discoveries | Article | [19] | 2016 | 0 | 1 | 1 | 1 | 0 | 1 | 0 | 0 | |
| 26 | Advanced light microscopy core facilities: Balancing service, science and career | Article | [53] | 2016 | 1 | 1 | 1 | 1 | 0 | 0 | 0 | 0 | |
| 27 | Institutional core facilities: prerequisite for breakthroughs in the life sciences: core facilities play an increasingly important role in biomedical research by providing scientists access to sophisticated technology and expertise | Article | [8] | 2016 | 1 | 1 | 1 | 0 | 0 | 1 | 1 | 1 | |
| 28 | Metrics for success: strategies for enabling core facility performance and assessing outcomes | Article | [22] | 2016 | 1 | 1 | 1 | 0 | 1 | 0 | 1 | 0 | |
| 29 | The relevance of the multi-user laboratory for biology studies for scientific production at UFSC: perception of CCB postgraduate coordinators | Article | [31] | 2017 | 0 | 1 | 0 | 1 | 1 | 0 | 0 | 0 | |
| 30 | Challenges and opportunities for biological mass spectrometry core facilities in the developing world. | Article | [24] | 2018 | 1 | 1 | 1 | 1 | 1 | 0 | 1 | 0 | |
| 31 | Building a sustainable portfolio of core facilities: a case study | Article | [20]. | 2018 | 1 | 1 | 0 | 1 | 1 | 1 | 1 | 0 | |
| 32 | Pathways for the Co-creation of Service in Academia: An Ethnographic Analysis of Epistemic Cultures in Japanese Public Shared Core Facilities. | Article | [54] | 2018 | 1 | 0 | 1 | 0 | 1 | 0 | 0 | 0 | |
| 33 | Operational and fiscal management of core facilities: a survey of chief research officers | Article | [55] | 2019 | 1 | 0 | 1 | 0 | 0 | 0 | 0 | 0 | |
| 34 | Survey on scientific shared resource rigor and reproducibility | Article | [25] | 2019 | 1 | 1 | 1 | 0 | 0 | 0 | 0 | 0 | |
| 35 | One step ahead: innovation in core facilities | Article | [14] | 2019 | 0 | 1 | 1 | 0 | 1 | 0 | 1 | 1 | |
| 36 | Rector Ordinance No. 432, of April 16, 2019 - creation of Relam-UFU - Multiuser Laboratory Network of the Federal University of Uberlândia | Internal Documents | [44] | 2019 | 1 | 0 | 1 | 0 | 0 | 1 | 0 | 0 | |
| 37 | Bioinformatics core survey highlights the challenges facing data analysis facilities | Article | [56] | 2020 | 1 | 0 | 1 | 0 | 1 | 0 | 0 | 0 | |
| 38 | Building a quality management system in a core facility: a genomics core case study | Article | [26] | 2020 | 1 | 1 | 1 | 0 | 1 | 0 | 1 | 0 | |
| 39 | A survey of research quality in core facilities | Article | [43] | 2020 | 1 | 1 | 0 | 0 | 1 | 1 | 0 | 1 | |
| 40 | Impact of Scientific Platforms on Research Success | Article | [57] | 2020 | 1 | 1 | 1 | 1 | 0 | 0 | 0 | 0 | |
| 41 | To buy or to lease: The advantages and costs of leasing versus buying scientific instruments for academic core facilities | Article | [58] | 2020 | 1 | 0 | 1 | 1 | 1 | 0 | 0 | 0 | |
| 42 | A novel paradigm for expert core facility staff training Trends in Cell Biology | Article | [59] | 2020 | 0 | 1 | 0 | 1 | 0 | 0 | 0 | 0 | |
| 43 | Lessons, insights and newly developed tools emerging from behavioral phenotyping core facilities. | Article | [9] | 2020 | 1 | 1 | 0 | 0 | 0 | 1 | 1 | 0 | |
| 44 | An international survey of training needs and career paths of core facility staff | Article | [10] | 2021 | 0 | 0 | 0 | 0 | 0 | 0 | 1 | 0 | |
| 45 | Reopening during the unprecedented: the Association of Biomolecular Resource Facilities Community Coronavirus Disease 2019 Pandemic Response. part 2: efforts to effectively ramp up core facility activities | Article | [60] | 2021 | 1 | 1 | 1 | 1 | 1 | 0 | 0 | 0 | |
| 46 | Towards best practices in research: Role of academic core facilities | Article | [61] | 2021 | 1 | 1 | 1 | 1 | 0 | 0 | 0 | 1 | |
| 47 | Data Management Tools to Measure the Impact of Core Facilities | Article | [62] | 2021 | 0 | 0 | 0 | 0 | 0 | 0 | 0 | 0 | |
| 48 | Public Call MCTI/FINEP/FNDCT/CT-INFRA/National Multiuser Centers 2022 | Call for proposals | [28] | 2022 | 1 | 1 | 1 | 1 | 0 | 1 | 0 | 1 | |
| 49 | Assessing and Improving Research Quality in Core Facilities | Article | [63] | 2022 | 1 | 1 | 1 | 1 | 0 | 0 | 0 | 0 | |
| 50 | How tech‐savvy employees make the difference in core facilities: Recognizing core facility expertise with dedicated career tracks | Article | [64] | 2022 | 0 | 1 | 0 | 1 | 0 | 0 | 0 | 0 | |
| 51 | CNSP- Canadá – executive summary2022. | Internal Documents | [65] | 2022 | 0 | 1 | 1 | 1 | 0 | 1 | 1 | 1 | |
| 52 | Research Infrastructure Core Facilities at Research Centers in Minority Institutions: Part I—Research Resources Management, Operation, and Best Practices | Article | [66] | 2022 | 1 | 1 | 1 | 0 | 0 | 0 | 0 | 0 | |
| 53 | Call for Multiuser Equipment for Scientific Use - 2022 | Call for proposals | [29] | 2022 | 1 | 1 | 1 | 1 | 0 | 0 | 0 | 0 | |
| 54 | Acknowledging and citing core facilities: Key contributions to data lifecycle should be recognised in the scientific literature | Article | [12] | 2022 | 1 | 1 | 1 | 1 | 0 | 0 | 0 | 0 | |
| 55 | Branded’ microscopy core facilities–Mutually beneficial partnerships between academia and industry | Article | [67] | 2022 | 1 | 1 | 1 | 1 | 0 | 0 | 0 | 0 | |
| 56 | FAPEMIG Call 04/2023 Technology Centers and Infrastructure for Research at UEMG and UNIMONTES22 | Call for proposals | [30] | 2023 | 1 | 0 | 1 | 0 | 1 | 1 | 0 | 0 | |
| 57 | Challenges and opportunities for bioimage analysis core-facilities | Article | [13] | 2023 | 0 | 0 | 1 | 0 | 0 | 0 | 0 | 0 | |
| 58 | Elevating the Educational Mission of “Full-Service” Core Facilities through Formal Biotechnology Workshops | Article | [33] | 2023 | 1 | 1 | 1 | 1 | 0 | 1 | 0 | 1 | |
| 59 | Bringing science back to the core: A financially sustainable model for core facilities | Article | [68] | 2023 | 1 | 1 | 1 | 1 | 1 | 0 | 0 | 0 | |
| 60 | Internal Regulations of the Multiuser Laboratory for Biology Studies (LAMEB), linked to the Center for Biological Sciences (CCB) of the Federal University of Santa Catarina (UFSC). | Internal Documents | [32) | 2023 | 1 | 1 | 0 | 1 | 1 | 1 | 0 | 1 | |
| 61 | Can you keep up? The challenges for research institutes and core facilities in scouting and adopting new technologies | Article | [2] | 2024 | 1 | 1 | 1 | 1 | 1 | 1 | 0 | 1 | |
| 62 | More than just ‘added value’: The perils of not establishing shared core facilities in resource‐constrained communities | Article | [1] | 2024 | 1 | 1 | 1 | 1 | 1 | 1 | 0 | 0 | |
|  | A= EQUIPMENT AND TECHNOLOGIES; B= SERVICE PROVISION; C= CENTRALIZED AND SHARED RESOURCES; D= SPECIALIZED TECHNICAL TEAM; | | | | | | | | | | | |  |
|  | E= ECONOMY; F= COLLABORATION and INTERDISCIPLINARITY; G= BUSINESS POSTURE; H= DEVELOPMENT OF NEW TECHNOLOGIES | | | | | | | | | | | |  |

**Source:** Prepared by the authors, based on information obtained in the analytical review, in April 2024
